# Cortical Thinning in Temporo-Parietal Junction (TPJ) in Non-Affective First-Episode of Psychosis Patients with Persistent Negative Symptoms

**DOI:** 10.1371/journal.pone.0101372

**Published:** 2014-06-30

**Authors:** Michael Bodnar, Cindy L. Hovington, Lisa Buchy, Ashok K. Malla, Ridha Joober, Martin Lepage

**Affiliations:** 1 Prevention and Early Intervention Program for Psychoses (PEPP – Montreal), Douglas Mental Health University Institute, Montreal, Canada; 2 Department of Psychology, McGill University, Montreal, Canada; 3 Department of Neurology & Neurosurgery, McGill University, Montreal, Canada; 4 Department of Psychiatry, McGill University, Montreal, Canada; Institute of Automation, Chinese Academy of Sciences, China

## Abstract

**Background:**

Negative symptoms represent an unmet therapeutic need in many patients with schizophrenia. In an extension to our previous voxel-based morphometry findings, we employed a more specific, vertex-based approach to explore cortical thinning in relation to persistent negative symptoms (PNS) in non-affective first-episode of psychosis (FEP) patients to advance our understanding of the pathophysiology of primary negative symptoms.

**Methods:**

This study included 62 non-affective FEP patients and 60 non-clinical controls; 16 patients were identified with PNS (i.e., at least 1 primary negative symptom at moderate or greater severity sustained for at least 6 consecutive months). Using cortical thickness analyses, we explored for differences between PNS and non-PNS patients as well as between each patient group and healthy controls; cut-off threshold was set at *p*<0.01, corrected for multiple comparisons.

**Results:**

A thinner cortex prominently in the right superior temporal gyrus extending into the temporo-parietal junction (TPJ), right parahippocampal gyrus, and left orbital frontal gyrus was identified in PNS patients vs. non-PNS patients. Compared with healthy controls, PNS patients showed a thinner cortex prominently in the right superior temporal gyrus, right parahippocampal gyrus, and right cingulate; non-PNS patients showed a thinner cortex prominently in the parahippocampal gyrus bi-laterally.

**Conclusion:**

Cortical thinning in the early stages of non-affective psychosis is present in the frontal and temporo-parietal regions in patients with PNS. With these brain regions strongly related to social cognitive functioning, our finding suggests a potential link between primary negative symptoms and social cognitive deficits through common brain etiologies.

## Introduction

Cortical thinning in fronto-temporal regions has become a well-documented finding in schizophrenia [1], [2]. However, with the pivotal confounds associated with illness chronicity more recent studies have turned to exploring for morphological abnormalities in first-episode of psychosis (FEP) samples [3]–[5]. Although studies have shown cortical thinning related to symptomology during these early stages of illness, the relationship with negative symptoms remains vague with some studies identifying an association [6]–[8] and others not [3], [9]. The ambiguity of these findings may be due to the fact that not all studies explicitly explored primary negative symptoms.

Primary and enduring negative symptoms are symptoms intrinsic to schizophrenia [10] that are readily studied in people with either deficit syndrome (DS) or persistent negative symptoms (PNS). For DS, 2 out of 6 items on the Schedule for the Deficit Syndrome (SDS) [11] need to be present for a minimum of 12 months and can only be measured using the SDS [10], [12]. In contrast, for PNS, only 1 item of the 6 on the needs to be present for a minimum of 6 months; symptoms that can be measured using ratings scales other than the SDS (e.g., SANS or PANSS). Furthermore, PNS can easily characterized in FEP samples, removing such potential confounds such as illness or medication chronicity. These key characteristics have lead to a recent increase in studies exploring primary negative symptoms in PNS [10], [12]–[18].

As an extension to our VBM study exploring PNS in a FEP sample [15], we wanted to see if cortical thickness analyses would identify the same regions of interest (right frontal medial-orbital and right parahippocampal gyri). Moreover, since cortical thickness has not been used to examine PNS in FEP patients, we set out to determine if other regions of interest could be identified using this more precise technique [19]. At the methodological level, VBM analyses capture the volume of structures by the totality of voxels it encompasses or by examining gray matter density; in contrast, cortical thickness analyses examine MRIs at a subvoxel level to provide a direct measurement in millimeters of gray matter morphology, an anatomically more meaningful measure reflecting cortical laminar structure and integrity. With VBM and cortical thickness becoming easily accessible imaging techniques, comparability of results between the two methods is a topic of great interest [19]–[22] and has been cited as a necessary step when investigating the pathophysiology of disorders such as schizophrenia [23].

## Materials and Methods

### 2.1 Participants & Treatment Setting

All patients were recruited and treated through the Prevention and Early Intervention Program for Psychoses (PEPP-Montreal), a specialized early intervention service at the Douglas Mental Health University Institute serving a local catchment area in Montreal, Canada. People aged 14 to 35 years experiencing an affective or non-affective first-episode of psychosis who had not previously taken antipsychotic medication for more than one month with an IQ higher than 70 were consecutively admitted to the program as either in- or out-patients. For complete program details see [24] or http://www.douglas.qc.ca/pages/view?section_id=165&locale=en. Only those with a non-affective diagnosis who were over the age of 18 years were included in the analysis.

Patients were identified as having ‘persistent negative symptoms’ if they had a global rating of moderate (value of 3) or more on at least one negative symptom (affective flattening, alogia, avolition-apathy, or anhedonia-asociality) as measured with the Scale for the Assessment of Negative Symptoms (SANS) [25]. Of note, if a global score of 3 or more was given on affective flattening and alogia entirely as a result of *inappropriate affect* and *poverty of content of speech*, respectively, these symptoms were not used in classifying PNS. Next, to ensure PNS were indeed primary negative symptoms, PNS patients had to have a global rating of mild (value of 2) or less on all positive symptoms as measured with the Scale for the Assessment of Positive Symptoms (SAPS) [26], a total score of 4 or less on the Calgary Depression Scale for Schizophrenia (CDSS) [27], and extrapyramidal symptoms that were absent or too mild to require treatment with anticholinergic medications. Finally, all scores had to be maintained for a period of at least 6 consecutive months (between month 6 and 12 after admission, in our case). See Hovington et al [12] for further details regarding the adapted criteria used for identifying PNS.

In all, 62 non-affective FEP patients were subsequently separated into two groups: PNS (n = 16, 25.8%) and non-PNS (n = 46, 74.2%). Among the 46 non-PNS patients, eight displayed PNS but were excluded from the PNS group because of clinically relevant positive (n = 6) and depressive symptoms (n = 2); none were excluded due to extrapyramidal symptoms. Diagnoses included: schizophrenia (PNS = 11; non-PNS = 33), schizoaffective disorder (PNS = 4; non-PNS = 7), schizophreniform disorder (non-PNS = 1), and psychosis NOS (PNS = 1; non-PNS = 5) according to the Structured Clinical Interview for DSM-IV [28] confirmed between two senior research psychiatrists (A.M. & R.J.).

Sixty non-clinical controls were recruited through advertisements in local newspapers and were included only if they had no current or past history of 1) any Axis I disorders, 2) any neurological diseases, 3) head trauma causing loss of consciousness, and 4) a first-degree family member suffering from schizophrenia or related schizophrenia spectrum psychosis.

### 2.2 Ethics Statement

All research was conducted according to the guidelines laid out by the Declaration of Helsinki, and was approved by the Research Ethics Board of the Douglas Mental Health University Institute and the McGill University Faculty of Medicine Research Ethics Board. All participants provided written informed consent prior to engaging in any research-related activity, and were free to withdraw from the study at any time; verbal consent was not considered adequate. Particular to the patients, for the collection and disposition of clinical-based data, if a client was under 18 years of age or deemed incapable to properly represent themselves, written informed consent was obtained from the next of kin, caretaker, or legal guardian. The capacity for individual clients to provide consent was determined by the individual treating team (psychiatrist, case manager, and clinical evaluator) and confirmed by either of the two senior staff psychiatrists (A.K.M. & R.J.). For the collection and disposition of the neuroimaging data, only those aged 18 years and over were recruited from the PEPP clinic, and only after obtaining written informed consent for the collection and disposition of clinical-based data. Finally, after a comprehensive description of the neuroimaging study was provided and the patient displayed a complete understanding, written informed consent was obtained.

### 2.3 Data Collection

#### 2.3.1 Symptom, Medication, and Socio-demographic Data

The SANS, SAPS, CDSS, and anticholinergic data were obtained at first assessment and at months 1, 2, 3, 6, 9, and 12 after first assessment; first assessment was conducted, on average, within one month after admission (in days; mean = 25.5, s.d. = 9.3, range = 4.8–51.0). Evaluators at PEPP have established an ICC of 0.74 on the SAPS and 0.71 on the SANS; all raters participated in inter-rater reliability sessions at least once a year to avoid rater drift. The type and dosage of antipsychotic taken were also recorded and subsequently converted into chlorpromazine equivalents [29]–[31]. Medication adherence, based on a 5-point scale ranging from 0 (never) to 4 (fully), was obtained from patients or, when possible, from family members; method was validated elsewhere [32]. Additionally, the following data were acquired at first assessment: education level (number of school years completed), Full Scale IQ with the Wechsler Adult Intelligence Scale [33], parental socio-economic status (SES) with the Hollingshead two-factor index [34], and handedness with the Edinburgh Handedness Inventory [35].

#### 2.3.2 MRI Data Acquisition

Scanning was carried out at the Montreal Neurological Institute on a 1.5 T Siemens whole body MRI system. Structural T1 volumes were acquired for each participant using a three-dimensional (3D) gradient echo pulse sequence with sagittal volume excitation (repetition time = 22 ms, echo time = 9.2 ms, flip angle = 30°, 180 1 mm contiguous sagittal slices). The rectangular field-of-view for the images was 256 mm (SI)×204 mm (AP). Patient groups did not differ as to when sessions took place past entry (weeks; PNS mean = 15.9, s.d. = 5.8; non-PNS mean = 19.9, s.d. = 7.8; t = 1.82, df = 60, *p* = 0.07).

### 2.4 Statistical Analyses

#### 2.4.1. Measurement of Cortical Thickness

MRIs were submitted to the CIVET processing pipeline (Version 1.1.9) (http://wiki.bic.mni.mcgill.ca/index.php/CIVET) [36], [37]. Native T1-weighted images were first registered to the ICBM152 template using linear transformation [38], [39] and simultaneously corrected for non-uniformity artifacts using N3 [40]. The transformed images were then segmented into grey matter, white matter, cerebral spinal fluid and background using a neural net classifier (INSECT) [37]. Grey matter and white matter surfaces were extracted using CLASP algorithm [41]–[43]. A spherical-mesh deformation algorithm was used to produce a surface mesh of 81 920 polygons (40 962 nodes or vertices) for each hemisphere. Nonlinear registration of both cortical surfaces to a high resolution average surface template generated from the ICBM152 data set was performed to establish inter-subject correspondence of vertices [44], [45]. Reverse linear transformation of volumes was performed to allow vertex-based corticometric (VBC) measurements in native space for each subject’s MRI [46]. The deformation algorithm first fits the white matter surface and then expands to the outer GM and cerebral spinal fluid intersection. From these surfaces, cortical thickness was computed in native space using the t-link method [47], which determines the linked distance between the inner and outer cortical surfaces at each of 40 962 vertices. Each participant’s cortical thickness map was subsequently blurred using a 20-mm full-width at half-maximum surface-based diffusion smoothing kernel [48].

Statistics were performed at all 40 962 vertices using three difference contrasts: PNS vs. non-PNS, PNS vs. Controls, and non-PNS vs. Controls. Total intracranial volume was not included as a covariate as cortical thickness and brain volume are poorly correlated [46], [49]. Statistical maps were thresholded and multiple comparisons were taken in to account using the false discovery rate procedure, with q = 0.05 [50]; results were considered significant at t = 2.64 (p<0.01).

#### 2.4.2 Whole-brain Tissue Volumes

Finally, whole-brain GM, WM, and CSF volumes were estimated using VBM8 (http://dbm.neuro.uni-jena.de/vbm/download/) for each participant and were summed for an estimation of total intracranial volume (TIV); the four volumes were compared among the three groups using an ANOVA (post-hoc Tukey’s HSD test).

#### 2.4.3 Behavioral Analyses

Among the three groups, age at scan, education level, and Full Scale IQ were compared using a one-way ANOVA (post-hoc Tukey’s HSD test), parental SES with a Kruskall-Wallis H-test (post-hoc Mann-Whitney U-test), and sex (male vs. female) and handedness (right vs. other) with cross tabulation and Chi-square tests. Between patient groups, independent t-tests were used to compare antipsychotic dosage and symptom totals and Mann-Whitney U-tests to compare medication adherence at first assessment, month 6, and month 12. CDSS ratings were log-transformed while SAPS ratings and antipsychotic total dosage were square-root transformed to achieve normal distribution; all other variables were normally distributed. All analyses were conducted using PASW Statistics 18 (SPSS Inc., 2009, Chicago, IL, USA) and were two-tailed with a critical *p*-value of 0.05.

## Results

### 3.1 Socio-demographic and Clinical Characteristics

The groups did not significantly differ in age, parental SES, sex, or handedness. PNS and non-PNS patients had fewer years of education and a lower Full Scale IQ compared to controls; the patient groups did not significantly differ (Table 1). Patient groups did not significantly differ in negative symptoms at first assessment but the PNS patients showed significantly higher totals at month 6 and 12, as expected. The two groups did not significantly differ in positive or depressive symptoms, total antipsychotic dosage (in chlorpromazine equivalents), and medication adherence at any time point (Table 2).

**Table 1:** Socio-demographic characteristics and whole-brain tissue volumes for PNS patients, non-PNS patients, and controls.

	PNS (n = 16)	non-PNS (n = 46)	Controls (n = 60)	*p*
**Socio-demographic variable**				
Age at scan (years)	24.2±4.3	23.7±3.4	24.8±3.3	0.285
Parental SES^a^	3.4±1.0	3.4±1.2	3.1±1.1	0.394
Education level^b^	11.2±2.0	12.1±2.6	14.4±2.5	<0.001
Full Scale IQ^c^	97.6±18.2	95.5±12.2	107.9±14.9	<0.001
Handed, Right/Other	12/4	40/6	55/5	0.193
Sex, Male/Female	13/3	32/14	40/20	0.529
**Whole-brain tissue volumes (ml)**				
Grey matter	624±56	643±60	658±71	0.161
White matter	605±65	596±64	618±71	0.264
Cerebral-spinal fluid	201±27	197±27	203±35	0.656
Total intracranial	1430±127	1437±121	1479±151	0.220

Abbreviations: PNS, persistent negative symptoms.

a Hollingshead parental socioeconomic status: 1 = highest and 5 = lowest.

b Education level measured as number of years completed; post-hoc tests revealed: PNS = non-PNS (*p* = 0.285); PNS < controls (*p*<0.001); non-PNS < controls (*p*<0.001).

c Full Scale IQ measured with the WAIS-III (data were available for only 58 controls); post-hoc tests revealed: PNS = non-PNS (*p* = 0.870); PNS < controls (*p* = 0.034); non-PNS < controls (*p*<0.001).

**Table 2:** Clinical characteristics for PNS patients and non-PNS patients.

	PNS (n = 16)	non-PNS (n = 46)	*p*
**Negative symptom total (SANS)**			
First Assessment	31.2±13.6	27.4±12.5	0.308
Month 6	30.1±11.3	16.6±11.7	<0.001
Month 12	30.3±14.7	14.9±10.0	<0.001
**Positive symptom total (SAPS)**			
First Assessment	34.5±10.7	35.0±17.9	0.909
Month 6	10.9±9.2	9.7±11.9	0.710
Month 12	14.7±14.2	10.5±17.9	0.399
**Depressive symptom total (CDSS)**			
First Assessment	4.1±4.3	4.9±5.2	0.552
Month 6	3.4±3.7	1.8±3.3	0.105
Month 12	1.9±2.5	1.9±3.2	0.920
**Antipsychotic dosage (mg/day)** a			
First Assessment	151.5±116.1	170.7±161.8	0.665
Month 6	178.4±164.9	198.7±199.6	0.717
Month 12	104.7±65.7	206.4±253.4	0.119
**Medication adherence** b			
First Assessment	3.3±1.5	3.2±1.5	0.591
Month 6	3.1±1.2	3.0±1.4	0.951
Month 12	2.3±1.9	3.2±1.5	0.126

Abbreviations: PNS, persistent negative symptoms; SANS, Scale for the Assessment of Negative Symptoms; SAPS, Scale for the Assessment of Positive Symptoms; CDSS, Calgary Depression Scale for Schizophrenia.

a Antipsychotic totals presented in chlorpromazine equivalents.

b Medication adherence: 0 (never adherent) to 4 (fully adherent).

### 3.2 Cortical Thickness

#### 3.2.1 PNS patients vs. non-PNS patients

**Figure 1:**
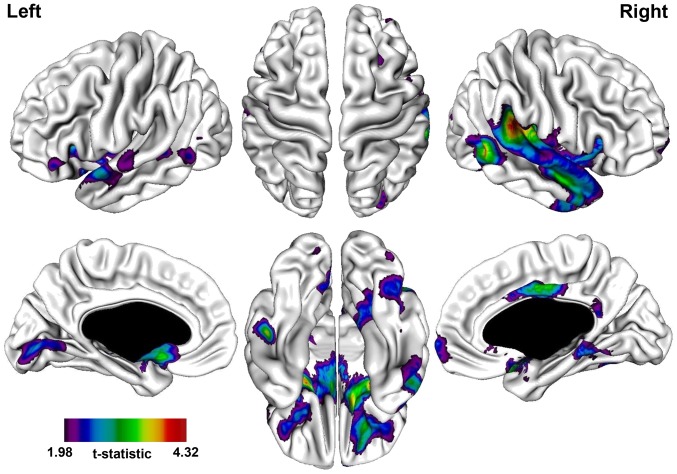
t-statistical brain maps showing cortical thinning in patients with persistent negative symptoms compared to patients without persistent negative symptoms. Most pronounced differences in the right temporo-parietal junction, right superior temporal gyrus, right parahippocampal gyrus, and left inferior frontal gyrus. The colour bar indicates the t-value. All areas shown exceed a FDR corrected statistical threshold of P<0.01.

A significantly thinner cortex in the PNS patients was observed in the following regions: bilateral frontal, temporal, fusiform, and occipital gyri, right parahippocampal gyrus, bi-lateral anterior cingulate, and right middle and posterior cingulate (Table 3; Figure 1). The most prominent difference was observed in the right superior temporal gyrus extending into the temporo-parietal junction (near the angular gyrus).

**Table 3:** Areas of cortical thinning in PNS patients compared to non-PNS patients.

Region (Brodmann Area)	Coordinates in MNI space			
	x	y	z	t-value
**Right hemisphere**				
Medial frontal gyrus (10)	6	66	−4	2.49
Orbital frontal gyrus (47)	20	32	−23	3.25
Anterior cingulate (24)	3	30	−7	2.13
Parahippocampal gyrus (34)	26	7	−17	4.19
Inferior temporal gyrus (20)	50	0	−34	3.09
Anterior/middle cingulate (24/23)	3	−3	38	3.34
Middle temporal gyrus (21)	50	−5	−20	3.30
Middle temporal gyrus (39)	56	−55	5	4.22
Superior temporal gyrus (41)	41	−34	17	4.32
Posterior cingulate (30)	3	−47	19	2.38
Fusiform gyrus (37)	42	−65	−16	2.54
Middle occipital gyrus (19)	30	−85	18	2.38
**Left hemisphere**				
Inferior frontal gyrus (47)	−54	35	−1	2.36
Middle frontal gyrus (11)	−23	27	−17	2.69
Subgenual cingulate (25)	−3	11	−10	3.39
Inferior frontal gyrus (47)	−19	8	−19	3.97
Superior temporal gyrus (22)	−59	3	−7	2.89
Fusiform gyrus (20)	−48	−28	−25	3.74
Middle temporal gyrus (22)	−51	−41	2	2.44
Middle temporal gyrus (21)	−56	−58	1	2.41
Middle temporal gyrus (39)	−49	−69	11	2.07
Cuneus (17)	−7	−83	2	2.80
Lingual gyrus (18)	−13	−88	−12	2.30

Abbreviations: PNS, persistent negative symptoms.

#### 3.2.2 PNS patients vs. Controls

**Figure 2:**
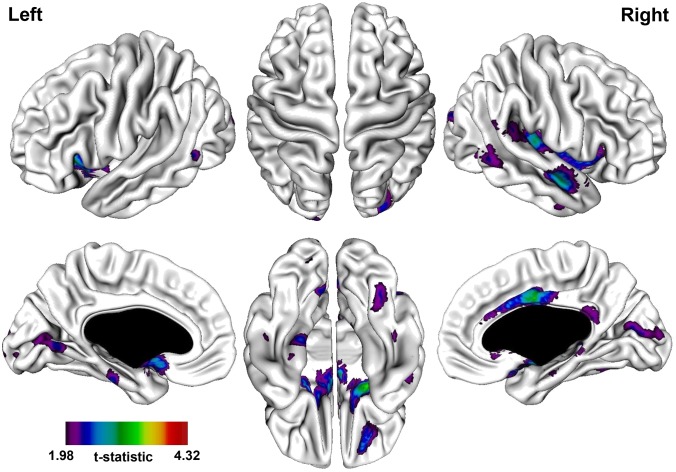
t-statistical brain maps showing cortical thinning in patients with persistent negative symptoms compared to healthy controls. Most pronounced differences in the right temporal gyrus, right parahippocampal gyrus, and right anterior/middle cingulate. The colour bar indicates the t-value. All areas shown exceed a FDR corrected statistical threshold of P<0.01.

A significantly thinner cortex in the PNS patients was observed in the following regions: bi-lateral frontal, temporal, fusiform, parahippocampal, and occipital gyri, bi-lateral anterior cingulate, and right middle and posterior cingulate (Table 4; Figure 2). The most prominent difference was observed in the right parahippocampal gyrus.

**Table 4:** Areas of cortical thinning in PNS patients compared to controls.

Region (Brodmann Area)	Coordinates in MNI space			
	x	y	z	t-value
**Right hemisphere**				
Middle frontal gyrus (11)	18	48	−20	2.66
Anterior cingulate (32)	3	23	−8	2.25
Parahippocampal gyrus (34)	22	5	−17	3.45
Inferior temporal gyrus (20)	49	−3	−32	2.38
Anterior/middle cingulate (24/23)	2	−4	35	3.23
Superior temporal gyrus (21)	52	−5	−15	2.98
Parahippocampal gyrus (28)	24	−18	−20	2.38
Fusiform gyrus (20)	43	−30	−20	2.19
Parahippocampal gyrus (27)	16	−36	−3	2.37
Posterior cingulate (30)	3	−45	22	2.34
Middle temporal gyrus (21)	58	−56	1	2.48
Fusiform gyrus (37)	37	−58	−16	2.36
Middle temporal gyrus (37)	45	−63	−2	2.64
Cuneus (18)	5	−78	12	2.41
Cuneus (19)	26	−91	21	2.47
**Left hemisphere**				
Anterior cingulate (25)	−4	10	−10	2.79
Inferior frontal gyrus (47)	−18	9	−19	2.79
Parahippocampal gyrus (36)	−29	−13	−30	2.25
Parahippocampal gyrus (35)	−22	−24	−26	2.52
Fusiform gyrus (20)	−48	−28	−26	2.11
Middle temporal gyrus (21)	−55	−56	2	2.39
Cuneus (30)	−6	−66	4	2.42
Lingual gyrus (18)	−15	−84	−15	2.01
Middle occipital gyrus (18)	−16	−102	11	2.51

Abbreviations: PNS, persistent negative symptoms.

#### 3.2.3 non-PNS patients vs. Controls

**Figure 3:**
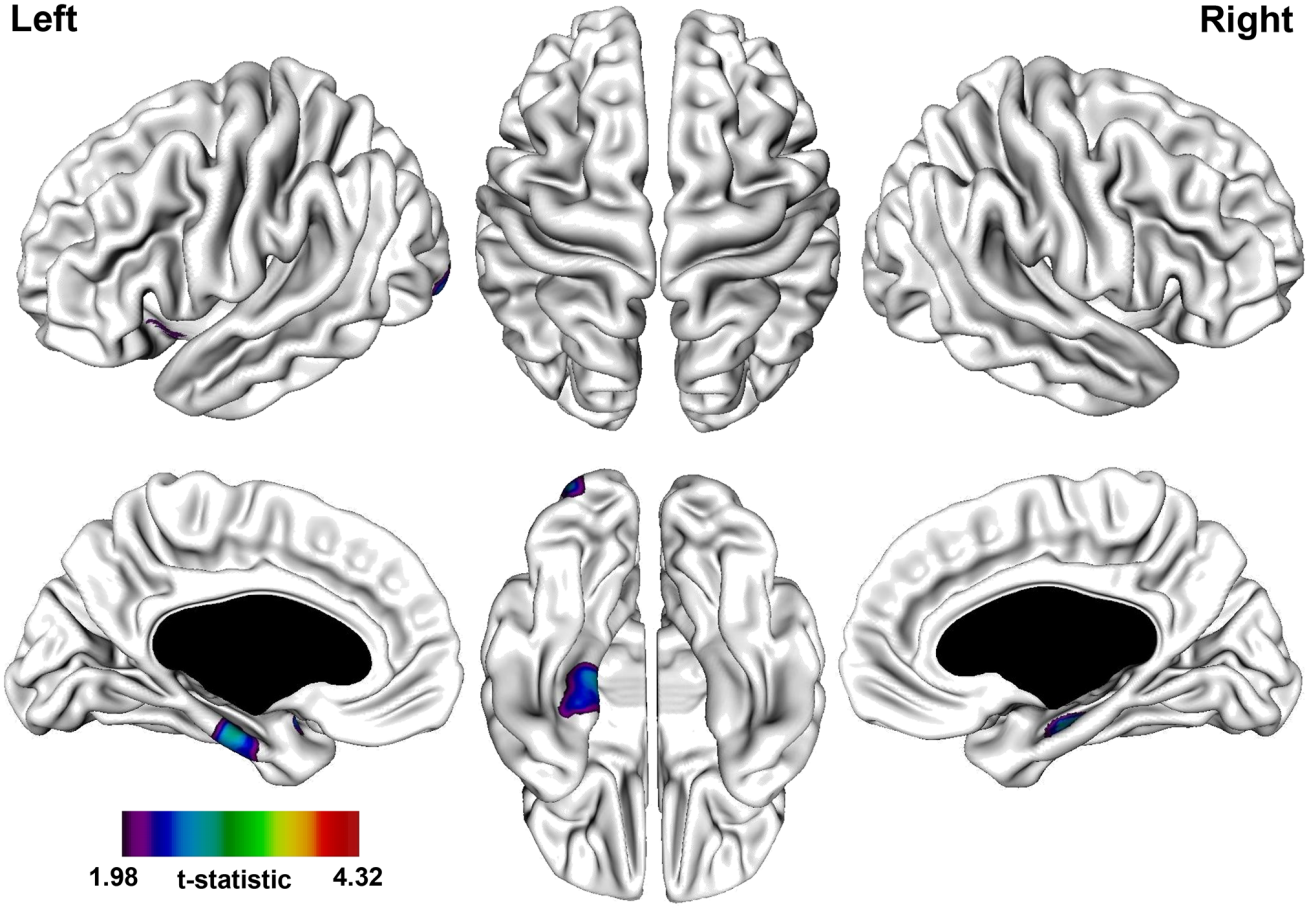
t-statistical brain maps showing cortical thinning in patients without persistent negative symptoms compared to healthy controls. Most pronounced difference in the parahippocampal gyrus bi-laterally. The colour bar indicates the t-value. All areas shown exceed a FDR corrected statistical threshold of P<0.01.

A significantly thinner cortex in the non-PNS patients was observed in the following regions: bi-lateral parahippocampal gyrus, left superior temporal gyrus, and left inferior occipital gyrus (Table 5; Figure 3). The most prominent differences were observed in the bi-lateral parahippocampal gyrus.

**Table 5:** Areas of cortical thinning in non-PNS patients compared to controls.

Region (Brodmann Area)	Coordinates in MNI space			
	x	y	z	t-value
**Right hemisphere**				
Parahippocampal gyrus (34)	30	−19	−19	2.92
**Left hemisphere**				
Superior temporal gyrus (38)	−34	7	−21	2.64
Parahippocampal gyrus (28)	−19	−19	−23	2.92
Inferior occipital gyrus (18)	−28	−96	−13	2.72

Abbreviations: PNS, persistent negative symptoms.

## Discussion

The present study used cortical thickness - a more precise method that directly measures gray matter morphology in millimeters reflecting cortical laminar structure and integrity - to explore the neural correlates of persistent negative symptoms (PNS) in non-affective first-episode of psychosis (FEP) patients using a well-established criteria for PNS.

We found a thinner cortex (less grey matter) in the right medial-orbital gyrus and right parahippocampal gyrus in the PNS patients compared to non-PNS patients, supporting of our previous VBM findings [15]. However, we were also able to identify cortical thinning in the PNS patients in additional frontal (cingulate cortex bilaterally) and temporal (temporal gyrus and fusiform gyrus bilaterally) regions, with the largest area of thinning extending into the temporo-parietal junction (TPJ). As well, when compared to controls, PNS patients showed greater cortical thinning overall, more notably in the anterior cingulate bilaterally, temporal gyrus bilaterally, and left parahippocampal gyrus. Given these findings, it is clear that the neural correlates of PNS involve multiple cortical and subcortical regions.

### 4.1 Negative Symptoms and the Temporal Lobe

#### 4.1.1 Superior Temporal Gyrus and Temporo-parietal Junction

To date, this is the first study, to the best of our knowledge, to identify a thinner cortex in the superior temporal gyrus (STG) extending into the temporo-parietal junction (TPJ) in FEP patients with PNS.

Previous imaging studies have identified significantly less grey matter in the STG in relation to primary and enduring negative symptoms [6], [8], [51]–[53]. However, other studies [54], [55] including our previous VBM analysis [15] did not demonstrate this relationship. Moreover, other studies have shown a correlation with positive symptoms [56], [57], supporting its significant role in auditory and language processing [58], [59]. The involvement of the STG as related to PNS appears somewhat unclear [60]. Alternatively, cortical thinning in the STG extending into the TPJ could be related to the social cognitive deficits that define non-affective psychoses, such as schizophrenia [61].

People with PNS are defined by a lack of social skills from not smiling (flat affect) to not talking (poverty of speech) with many withdrawing from society (asociality) or choosing not to engage in everyday activities (avolition). These “missing” social skills are innate to their presentation of psychosis. Our strongest finding was in the right TPJ and this region has received increasing attention concerning its role in social cognition, empathy, and social salience. The TPJ has been associated with various social cognitive processes [62]–[65] with the right TPJ specifically related to the attribution of thoughts in others compared to the attribution of appearance or bodily-sensations about a person [66], [67]. The TPJ has also been implicated in identification and reorientation towards salient events in the sensory environment [68], [69]. However, one study demonstrated that TPJ activation is limited to choice deliberation about the nature of the upcoming decision in a social context rather than external social stimuli itself [70]. Nevertheless, the TPJ is believed to play a critical role in coordinating behavior in a dynamic, social environment. We as humans must be able to make adaptive socially-correct decisions in a social context and the TPJ is central to this ability [61], [70].

Of course, the TPJ has been generally associated with the positive symptoms of schizophrenia based on the plethora of studies that either evoke [71], [72] or disrupt [73]–[76] activation of the TPJ leading to the induction or alleviation of auditory-related symptoms, respectively. However, there is a new direction that suggests schizophrenia as a social communication disorder with the TPJ as a central structure of interest [61]. Our findings support this idea if we equate primary negative symptoms to a diminished social cognitive ability (flat affect, poverty of speech, asociality, or avolition). Needless to say, we know that structure size does not correlate with function, but a lack of grey matter in the STG and TPJ could help us better understand the relationship among negative symptoms, socializing, and psychosis. Moreover, it could point to a region of interest in developing new treatments for this with primary negative symptoms.

#### 4.1.2 Parahippocampus

Reduced grey matter in the parahippocampus has been identified as a consistent finding in schizophrenia [77]–[79] with these reductions associated with negative symptom severity [51], [52], [80], [81]. However, these associations have been left-lateralized [52] or bilateral [51], [80], [81]. In contrast, our analyses examining PNS patients vs. non-PNS patients identified a thinner cortex as well as a reduced volume specific to the right side [15]. Yet, PNS patients showed a thinner cortex bilaterally compared to controls. As such, the association of negative symptoms with laterality is unclear or perhaps associations exist related to specific negative symptoms. For example, our group identified in FEP patients a significant correlation between higher social withdrawal ratings and reduced parahippocampal grey matter consistently on the right side [80], [81] whereas, in people with chronic schizophrenia, we revealed a significant positive correlation between flat affect ratings and parahippocampal activity bilaterally [82]. Although these studies did not explicitly investigate primary negative symptoms, a neurobiological association may exist with specific negative symptoms that was not explored in the abovementioned studies. Further investigations are needed to explore the neurobiological basis of individual negative symptoms.

### 4.2 Cortical Thickness Vs. Voxel-Based Morphometry (VBM)

Recent studies have emerged using both cortical thickness and VBM to study various populations including healthy-aging controls [19], Alzheimer’s [20], late-life depression [21], and schizophrenia [23]. In the study examining late-life depression, results revealed cortical thickness was more sensitive in detecting group differences than VBM [21]. Similarly, in the healthy-aging sample, the authors revealed both methods yield similar results but with cortical thickness more sensitive to grey matter decline [19]. Hutton et al elaborated on this by mentioning “[cortical thickness] is expected to be more sensitive than [VBM]… if there is a prior hypothesis that grey matter changes are mainly due to changes in cortical thickness and also if there is any correlation between the effect of interest and the total brain volume” [19]. Interestingly, for the Alzheimer’s study comparing posterior cortical atrophy, similar results were found using both techniques [20]. Finally, for the schizophrenia study, Palaniyappan and Liddle concluded, “while VBM may be more sensitive in identifying the regions with gray matter abnormalities, studies investigating the pathophysiology of illnesses such as schizophrenia are better informed when both [cortical thickness] and VBM analyses are performed concurrently” [23]. In fact, Hutton et al drew a similar conclusion stating that both techniques should be used together to better separate and understand the underlying grey matter changes.

From our analyses, the cortical thickness analysis appeared more sensitive than our VBM analysis [15] in detecting group differences regarding negative symptoms in non-affective FEP patients. What could count for these difference considering that the same sample was examined? First, we must reiterate the fact that cortical thickness specifically measures the cortex thickness in millimeters while VBM measures grey matter differences in local surface area and cortical folding [19]. This leads to the possible reasons why VBM fails to detect more grey matter differences related to: (1) the changes in the shape or displacement of structures during spatial normalization [83]–[86] or (2) the variability of gyrification [87] that has been shown to be present in schizophrenia [88]–[92]. Furthermore, the blurring of cortical thickness data takes place in a topologically correct manner along the cortical surface, whereas VBM blurring is 3-dimensional, meaning it does not respect boundaries between tissue classes, leading to an increased likelihood of diluting existing signal or misinterpreting boundary shift as signal [22].

Importantly, we must highlight that our previous VBM analysis used a statistical threshold of p<0.05, family-wise error (FWE) corrected for multiple comparisons [15] while the current cortical thickness analysis used a statistical threshold of p<0.01, false-discovery rate (FDR) corrected. Although FWE is prone to more false negatives and FDR (considered a less stringent correction than FWE) is prone to more false positives [93], studies examining these corrections (using VBM) have shown results to be similar [94], [95]. So, any observed differences in sensitivity between the techniques should not be solely attributed to the correction method employed. In addition, by using a cut-off of p<0.01 in the cortical thickness analysis we reduced the number of possible false positives and, effectively, the number of identifiable regions. Yet, more grey matter differences were still identified using this technique.

Taken together, it would appear that cortical thickness may be more sensitive in detecting grey matter anomalies in schizophrenia or related psychoses compared to VBM. This may help to explain why the cortical thickness analysis was able to detect more differences between all of the contrasts investigated and was able to detect more regions of interest related to primary negative symptoms. But for a more complete understanding of group differences in grey matter both techniques should be used to complement each other.

### 4.3 Conclusions

Our results along with previous studies investigating primary negative symptoms highlight neural abnormalities in two key regions: the frontal and temporal areas [6], [8], [15], [51], [52], [54] supporting the proposed prefronto-temporolimbic model of negative symptoms [60], [96]. Moreover, in the PNS patients, the largest area of cortical thinning was found in the right superior temporal gyrus extending into the temporo-parietal junction-core structures related to social cognitive functioning [61], [62]–[65]. With both social cognitive deficits and negative symptoms characterizing schizophrenia, this area could be explored further in the development of more effective treatments. In fact, treatments utilizing transcranial magnetic stimulation have targeted both the frontal and temporal areas, but the temporal region has been generally targeted in the hope of alleviating positive symptoms [76]. So, perhaps the temporo-parietal junction could be targeted in the hope of alleviating negative symptoms or even the social cognitive difficulties expressed in people with schizophrenia [61]. Finally, it must be stressed when investigating between-group grey matter differences in disorders like schizophrenia, multiple fully-automated techniques should be employed to provide a better understanding of the results [23].

### 4.4 Limitations

There are several limitations in our study. First, although we employed a first- episode sample in an attempt to reduce the effect of antipsychotic exposure on brain morphology [97], [98], the majority of patients were still treated with antipsychotics possibly affecting our results. Second, avolition and anhedonia were more prevalent than alogia and blunted affect in our sample and, as such, our results may not be generalizable to all primary negative symptoms. Furthermore, the categorical approach of this study did not allow us to specify which negative symptoms contributed the most to the structural differences identified; future studies need to examine these symptoms separately. Third, at the time of analysis, clinical data was only available for the first 12 months of treatment in our sample as it has been shown that PNS categorization is more consistent after the first year of treatment [17]. Fourth, our PNS patient group was relatively small, limiting the generalization (interpretation) of our results. Additionally, we could not examine whether the structural differences related to PNS were specific to one diagnosis or not because we were limited by the small number of patients with PNS that did not allow for any meaningful diagnosis specific between-group comparisons. Lastly, the non-PNS group were prescribed, on average, almost double the dosage of antipsychotics [in chlorpromazine equivalents (mg/day)] by month 12. Because treatment is determined on an individual basis at our clinic, we cannot provide any particular reason as to why the individuals of the PNS group were prescribed such a lower dosage. This is noteworthy as antipsychotics have been shown to affect brain morphology [97], [99]. However, with scanning completed 18 weeks, on average, after the start of antipsychotic treatment only minimal effects, if any at all, were expected regarding the frontal and temporal regions [100].
